# Leading causes of certification for blindness and partial sight in England & Wales

**DOI:** 10.1186/1471-2458-6-58

**Published:** 2006-03-08

**Authors:** Catey Bunce, Richard Wormald

**Affiliations:** 1 Research & Development, Moorfields Eye Hospital, City Road, London, UK

## Abstract

**Background:**

Prevention of visual impairment is an international priority agreed at the World Health Assembly of 2002- yet many countries lack contemporary data about incidence and causes from which priorities for prevention, treatment and management can be identified.

**Methods:**

Registration as blind or partially-sighted in England and Wales is voluntary and is initiated by certification by a consultant ophthalmologist. From all certificates completed during the year April 1999 to March 2000, the main cause of visual loss was ascertained where possible and here we present information on the leading causes observed and comment on changes in the three leading causes since the last analysis conducted for 1990–1991 data.

**Results:**

13788 people were certified as blind, 19107 were certified as partially sighted. The majority of certifications were in the older age groups. The most commonly recorded main cause of certifications for both blindness (57.2 %) and partial sight (56 %) was degeneration of the macula and posterior pole which largely comprises age-related macular degeneration. Glaucoma and diabetic retinopathy were the next most commonly recorded main causes. Overall, the age specific incidence of all three leading causes has increased since 1990–1991 – with changes in diabetic retinopathy being the most marked – particularly in the over 65's where figures have more than doubled.

**Conclusion:**

The numbers of individuals per 100,000 population being certified blind or partially sighted due to the three leading causes – AMD, diabetic retinopathy and glaucoma have increased since 1990. This may to some extent be explained by improved ascertainment. The process of registration for severe visual impairment in England and Wales is currently undergoing review. Efforts must be made to ensure that routine collection of data on causes of severe visual impairment is continued, particularly in this age of improved technology, to allow such trends to be monitored and changes in policy to be informed.

## Background

The number of blind people in Britain has been counted since 1851 and reports on the causes of low vision in England and Wales began in 1950 [1-6]. From the mid 1930's designated forms (BD8) signed by ophthalmologists were required in order to certify someone as blind or visually impaired. From 1991 the BD8 was a five-part form. Parts 1–4 were sent to the Local Authority Social services department who were responsible for adding the eligible person to the register. Part 5 was an anonymous epidemiological return containing data on the cause of visual impairment, which was sent to the Office of Population Censuses and Surveys (OPCS) {now known as the Office of National Statistics (ONS)}. The last analysis for all age groups was conducted by OPCS for the year ending March 1991 [7]. Here, we present findings from an analysis of all BD8 part 5's completed during the year April 1999–March 2000 and compare these with results form the 1991 analysis.

Table 1 shows the definitions of blindness and partial sight. These apply to the function of the better eye; people with good vision in one eye are not eligible for certification. It is important to note also that people are recommended for BD8 certification usually because they have irremediable blindness or partial sight – treatable causes of visual impairment are less likely to feature in the certified population eg cataract. Finally, it is important to note that in England and Wales, people are entitled to refuse the offer of certification and there is no statutory requirement for it to be offered.

## Methods

In 2003, all BD8 part 5 certificates completed during the year April 1999 to March 2000 in England and Wales were transferred from ONS to the Research and Development Department at Moorfields Eye Hospital after the specification and agreement of a research contract. Data were transferred from the paper certificates onto PC and coded using ICD-9 for ready comparison with the analysis conducted on the 1990–1991 data [8]. Data entry for five per cent the forms was duplicated to check for frequency of transcription and coding errors. Comparison with the data for 1990–1991 was made by computing the number of certifications per head of population.

## Results

Table 2 shows the numbers of people newly registered in England and Wales as reported to the DH by the Local Authority Social Services department (form SSDA902) and from Local Government Data Unit – Wales and the numbers of certificates received at ONS during the period April 1999 – March 2000. The figures are very similar – sixty five (in total) extra certificates were received by ONS for blindness registrations than were added to the register during 1999/2000 and for every 100 registrations for partial sight made during 103 certificates were received by ONS. Reasons for the excess include death of the registree before registration was taken up, subsequent decision not to be registered and incomplete data for registration supplied by the local authorities.

The numbers of certificates received by age and sex are presented in Table 3. We received 34410 BD8 certificates dated between April 1999 and March 2000, of whom 13788 were people certified as blind and 19107 partially sighted; 1515 (4.4 %) of the forms did not indicate whether the individual certified was blind or partially sighted. The majority of the certifications were in the older age groups; 83 % of the blind and 82 % of the partial sight certificates were completed for people aged 65 years and above. Between the ages of 0 and 64, 55 % of the blind certifications were for males but after the age of 65 the sex distribution was reversed with 64 % of blind certifications being female. A similar pattern was seen with partial sight – between the ages of 0–64, 51 % of partial certifications were for males but after the age of 65 67 % of partial sight certifications were for females.

**Figure 1:**
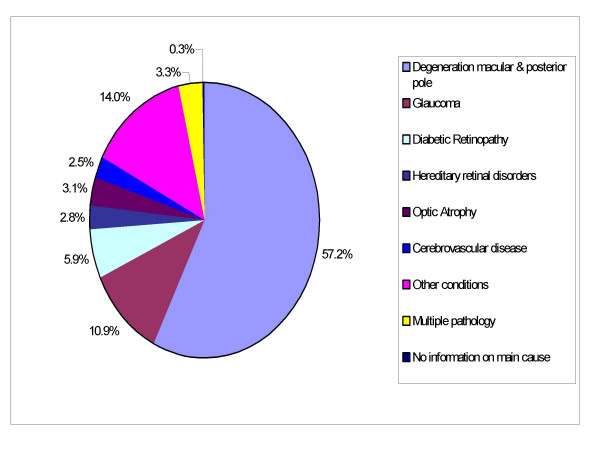
Causes of certifications for blindness in England and Wales April 1999 – March 2000.

Figure 1 shows the relative percentages of the leading causes of blindness certification. The most commonly recorded main cause of certification for blindness was degeneration of the macula and posterior pole (ICD 362.5) (57.2 %), which largely comprises age-related macular degeneration. Glaucoma (10.9 %), diabetic retinopathy (5.9 %), optic atrophy (3.1 %), hereditary retinal disorders (2.8 %), and cerebrovascular disease/accidents (2.5 %) were the next most frequently occurring causes of certification for blindness. If taken together, these causes accounted for over 80 % of blindness certifications during the year.

**Figure 2:**
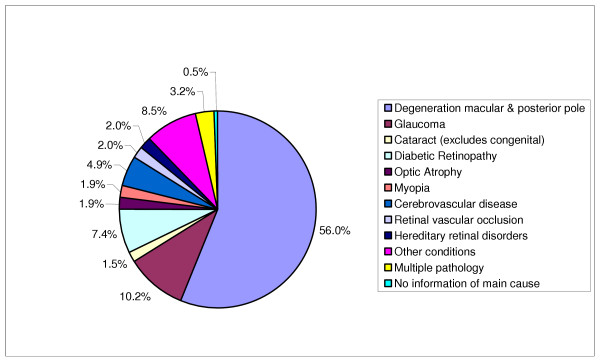
Causes of certifications for partial sight in England and Wales April 1999–March 2000.

Figure 2 shows the relative percentages of the leading causes of partial sight certification. As for blindness, the most commonly recorded main cause of certification for partial sight was degeneration of the macula and posterior pole (56%). Glaucoma (10.2 %), diabetic retinopathy (7.4%), cerebrovascular disease (4.9%), hereditary retinal disorders (2%), and optic atrophy (1.9%) were frequently occurring causes of certification for partial sight, as for blindness. Myopia and retinal vascular occlusions, however, which did not feature as a leading cause of blindness certification were the main causes for respectively 1.9% and 2 % of partial sight certifications in England & Wales in April 1999 to March 2000.

Table 4 shows the number of certifications (for blindness and partial sight combined) due to the three most common causes, AMD, diabetic retinopathy and glaucoma per head of population in 1999–2000 and similar figures for 1990/91. Overall there have been increases in all three – but changes are most marked for diabetic retinopathy. Table 4 shows fairly modest increases in certifications due to diabetes in the younger age groups, but in groups aged 65 and over, figures have more than doubled. Increases are seen in the older age groups for AMD but slight decreases in the 0–15 and 16–64 age group. For glaucoma, figures have remained similar or decreased in most age groups – the overall slight increase appears to be due to an increase in the number of individuals surviving beyond 85.

## Discussion

We present here the analysis of a very substantial national data set which is not based on sampling methodology and which report incidence of new cases of certification for visual impairment. We have for comparison, analysis of a previous data set collected nine years previously using essentially the same methods. Since this is not a sample survey, it is not appropriate to use sampling theory in the analysis and observed differences are actual not estimates. Biases clearly exist as to who and who does not become certified and it must be remembered that these data are hospital not population based since an individual has to access the hospital eye service to be seen by a consultant ophthalmologist. Essentially the same biases will have occurred at both time points and the same forms and eligibility criteria were in place. There may have been some drift in the threshold for certification by Consultants over time and this is most likely to have been a lowering of threshold. Over the period there have been a number of campaigns by agencies representing the interests of the visually impaired to highlight the importance of the registration process in facilitating the delivery of Social Service support to those who need it and there have been no formal audits published on eligibility for registration to our knowledge. We cannot know the extent to which this has happened because there is no objective information on visual function on Part 5 of the old BD8 form. This may account for some of the observed increase in registration. However, it is not obvious how this might be disease specific and one might expect such an increase to occur across all causes.

The population has aged considerably even in 9 years however we have provided age specific estimates to allow assessment of this. The population change may have had a differential effect by cause since AMD becomes exceedingly common in the very elderly [9].

None of this is likely to explain the near doubling of incidence of certifiable sight loss due to diabetic retinopathy in people over 65. This finding is of importance since it is contrary to what one might hope and expect with increased efforts being made for the detection and treatment of the condition. One explanation is that diabetics themselves are living longer but remain at risk of the disabling consequences of the disease.

Is an increase in diabetic blindness plausible? There have been increases observed for other diabetic complications such as diabetic nephropathy. UK data has suggested a doubling in incidence of childhood- onset type 1 diabetes between 1966 and 2000 [10]. The prevalence of type II diabetes is increasing; overweight/obesity being the single most important predictive factor for the development of diabetes [11].

Is an increase in AMD blindness likely or possible? There was an increase in 1990–91 as compared to previous years which is present despite the possible effects of previous miscoding [12]. If age effects have been adequately controlled for, there may yet be an absolute increase in the incidence of the disease. The only modifiable risk factor for the disease convincingly demonstrated from epidemiological studies is tobacco smoking [13]. Changes in the patterns of smoking over previous years, particularly the enormous increase in the prevalence of cigarette consumption after the Second World War may be emerging in increasing incidence of the disease.

Much criticism has been directed towards the validity and coverage of the data collected during BD8 certification [14-17]. It is estimated that approximately 53 % of eligible patients have not been registered blind or partially sighted despite consultation with an ophthalmologist. However, it must be noted that cross sectional studies are by their nature bound to detect under-registration because there is often a necessary delay between the onset of certifiable visual loss and the offer of registration. Individuals need time to come to terms with their loss of vision and consultants need time to determine whether an individual is certifiable. One should not underestimate the distress faced by an individual when told that their condition is certifiable for registration as blind or partially sighted. Measures of vision show variability – visual acuity can fluctuate in an individual with diabetes, and the point at which someone becomes certifiable due to visual field loss in glaucoma is not always easy to determine. These figures can surely be useful as indicators or minimum estimates of the incidence of severe sight loss in the population for planning preventive health care strategies and prioritising research particularly for irreversible causes.

The WHO stresses the importance of collecting within-country data on causes of visual blindness for use in priority-setting and resource allocation [18]. The future with regards analysis of data on the causes of visual impairment in England and Wales is unclear.

At the end of October 2000, a bulletin was circulated to all Directors of Social Services in England and Chief Executives of Health Authorities and Trusts by the Department of Health (DH) indicating that the DH no longer required the part 5 (the epidemiological return) of the form to be sent to ONS and that part 5 would be omitted for future reprint. In November 2003, following consultation with service users and key stakeholders, form BD8 was replaced by the Certificate of Vision Impairment 2003 in England – the form is currently undergoing trial and there is no commitment from the Department of Health for future analysis of data on causes of severe visual loss. A similar situation exists in Wales although their CVI has yet to be launched.

## Conclusion

This analysis, whilst imperfect, strongly suggests increases in the three main causes of sight loss in England and Wales. Each of these causes has a massive impact on the quality of life of the sight impaired but options for prevention and treatment exist for all of them. Surely in these times of improved technology, now is the time to improve the collection of good quality data on causes of visual impairment and establish an ongoing monitoring system.

## Competing interests

The author(s) declare that they have no competing interests.

## Authors' contributions

CB did the statistical analysis and wrote the first draft of the paper. Both authors contributed to the final version. CB and RW act as guarantors.

## Pre-publication history

The pre-publication history for this paper can be accessed here:



## Figures and Tables

**Table 1:** Definitions of blindness and partial sight used on BD8 certificates (apply to the better eye)

**Blindness**		**Partial Sight**	
**Legal definition**	so blind that they cannot do any work for which eyesight is essential	**Definition***(there is no legal definition)*	substantially and permanently handicapped by defective vision caused by congenital defect, illness or injury
**Recommendations:**	worse than 3/60 (corrected visual acuity) or worse than 6/60 with very contracted visual fields or 6/60 or above with a very contracted visual field especially in the lower part of the field	**Recommendations**	3/60 or 6/60 with full visual field or 6/24 or worse with moderate constriction of visual field or 6/18 or better with gross visual field defects

**Table 2:** Comparison of registration and certification for blindness and partial sight in England and Wales for April 1999–March 2000

	Total
**Blind**	
New registrations with Local Authority Social Services departments in England and Wales*	13723
BD8 (part 5) certificates received by ONS	13788
**Partially Sighted**	
New registrations with Local Authority Social Services departments in England and Wales*	18480
BD8 (part 5) certificates received by ONS	19107

* As reported to the DH by the Local Authority Social Services department (form SSDA902) and from Local Government Data Unit – Wales

**Table 3:** Blindness and partial sight in England & Wales; summary age-sex distribution of certifications; April 1999 – March 2000

	Total		Male		Female	
	Number	Column %	Number	Row %	Number	Row %
**Blind**						
0 – 64 years	1965	14	1087	55	860	44
65 years and above	11471	83	4034	35	7322	64
All ages *	13788	100	5245	38	8400	61
**Partially Sighted**						
0 – 64 years	2891	15	1476	51	1391	48
65 years and above	15725	82	5078	32	10501	67
All ages *	19107	100	6730	36	12195	62

* Data include age and sex unknown

**Table 4:** Age and sex specific rates of certification for severe visual impairment per 100,000 population due to diabetes, age related macular degeneration and glaucoma in 1990–91 and 1999–2000

	**Diabetes**			**AMD**		**Glaucoma**	
	Year	1990 – 1991	1999 – 2000	1990 – 1991	1999 – 2000	1990–1991	1999–2000
**All ages**	Both	1.99	3.84	24.41	31.78	5.43	5.91
	Male	1.75	3.44	15.28	19.96	4.59	5.31
	Female	2.15	4.15	31.82	42.44	6.00	6.32
0 – 15	Both	0.01	0.05	0.41	0.01	0.07	0.05
	Male	0.00	0.05	0.24	0.00	0.07	0.02
	Female	0.02	0.05	0.58	0.02	0.07	0.09
16 – 64	Both	1.26	2.05	1.48	1.01	0.55	0.55
	Male	1.31	2.26	1.29	0.91	0.65	0.76
	Female	1.19	1.84	1.61	1.10	0.41	0.34
65 – 74	Both	7.28	15.06	39.20	39.69	10.47	9.96
	Male	6.84	13.17	30.66	31.10	12.00	10.85
	Female	7.56	16.27	44.59	46.47	8.81	8.85
75 – 84	Both	8.27	17.08	213.50	251.53	47.85	44.14
	Male	6.93	15.03	184.86	208.76	52.18	50.98
	Female	8.65	18.18	221.81	275.70	44.34	38.75
85 and over	Both	3.92	11.02	541.07	699.02	100.31	113.62
	Male	5.41	13.46	560.96	682.94	135.20	152.14
	Female	3.13	9.86	516.43	697.37	85.29	97.59
